# Prospective study of predictors of vitamin D status and survival in patients with colorectal cancer

**DOI:** 10.1038/sj.bjc.6605262

**Published:** 2009-08-18

**Authors:** K Ng, B M Wolpin, J A Meyerhardt, K Wu, A T Chan, B W Hollis, E L Giovannucci, M J Stampfer, W C Willett, C S Fuchs

**Affiliations:** 1 Department of Medical Oncology, Dana-Farber Cancer Institute, 44 Binney Street, Boston, MA 02115, USA; 2 Department of Nutrition, Harvard School of Public Health, 655 Huntington Avenue, Boston, MA 02115, USA; 3 Department of Gastroenterology, Massachusetts General Hospital, 44 Fruit Street, Boston, MA 02114, USA; 4 Department of Pediatrics, Medical University of South Carolina, 173 Ashley Avenue, Charleston, SC 29425, USA; 5 Channing Laboratory, Department of Medicine, Brigham and Women's Hospital and Harvard Medical School, 181 Longwood Avenue, Boston, MA 02115, USA; 6 Department of Epidemiology, Harvard School of Public Health, 651 Huntington Avenue, Boston, MA 02115, USA

**Keywords:** vitamin D, colorectal cancer, epidemiology, diet and nutrition

## Abstract

**Background:**

In an earlier study, a 25-hydroxyvitamin D_3_ (25(OH)D) score calculated from known predictors of vitamin D status significantly predicted plasma levels of 25(OH)D and the risk of colorectal cancer, but the influence of the 25(OH)D score on survival after diagnosis is unknown.

**Materials and methods:**

We prospectively examined the influence of post-diagnosis predicted 25(OH)D levels on mortality among 1017 participants in the Nurses' Health Study and Health Professionals Follow-Up Study who were diagnosed with colorectal cancer from 1986 to 2004. Colorectal cancer-specific and overall mortality according to quintiles of predicted 25(OH)D levels were assessed. Cox proportional hazards models were used to calculate hazard ratios (HRs) adjusted for other risk factors of survival.

**Results:**

Higher predicted 25(OH)D levels were associated with a significant reduction in colorectal cancer-specific (*P* trend=0.02) and overall mortality (*P* trend=0.002). Compared with levels in the lowest quintile, participants with predicted 25(OH)D levels in the highest quintile had an adjusted HR of 0.50 (95% CI, 0.26–0.95) for cancer-specific mortality and 0.62 (95% CI, 0.42–0.93) for overall mortality.

**Conclusion:**

Higher predicted 25(OH)D levels after a diagnosis of colorectal cancer may be associated with improved survival. Further study of the vitamin D pathway in colorectal cancer is warranted.

The vitamin D hypothesis has received strong experimental support over the past two decades on the basis of the almost ubiquitous expression, in colon cancer cells, of vitamin D receptors (VDR) (Meggouh *et al*, 1991; Vandewalle *et al*, 1994) and 1-*α*-hydroxylase (Zehnder *et al*, 2001), which converts plasma 25-hydroxyvitamin D_3_ (25(OH)D) into 1,25-dihyroxycholecalciferol (1,25(OH)_2_D). Binding of VDR by 1,25(OH)_2_D leads to differentiation and apoptosis (Vandewalle *et al*, 1995; Diaz *et al*, 2000), and inhibition of proliferation (Scaglione-Sewell *et al*, 2000), angiogenesis (Iseki *et al*, 1999; Fernandez-Garcia *et al*, 2005), and metastatic potential (Evans *et al*, 2000; Lamprecht and Lipkin, 2001).

The best indicator of vitamin D status is plasma 25(OH)D level, as it reflects not only total vitamin D intake but also cholecalciferol production in the skin from type B ultraviolet (UV-B) radiation and hydroxylation of all sources of cholecalciferol in the liver. Prospective studies have shown that higher baseline plasma levels of 25(OH)D are associated with a significant reduction in the risk of colorectal cancer (Garland *et al*, 1989; Braun *et al*, 1995; Tangrea *et al*, 1997; Feskanich *et al*, 2004; Wactawski-Wende *et al*, 2006; Wu *et al*, 2007). A meta-analysis of five epidemiological studies found a 51% decrease in the risk of colorectal cancer associated with high plasma 25(OH)D levels (*P*<0.0001) (Gorham *et al*, 2007). In contrast, the influence of vitamin D on survival of patients with established colorectal cancer remains uncertain. In a previous study, we found that higher pre-diagnosis plasma 25(OH)D levels were associated with a significant improvement in overall survival among 304 colorectal cancer patients (Ng *et al*, 2008). However, this study was limited by the small number of patients who had available 25(OH)D plasma levels, and relied on a single measurement of 25(OH)D drawn at least 2 years before cancer diagnosis.

To evaluate the impact of vitamin D status after a diagnosis of colorectal cancer in a larger number of patients, we developed a model to predict plasma 25(OH)D levels. This 25(OH)D score takes into account the combined influence of the major determinants of vitamin D status (race as a surrogate of skin pigmentation, residential state as a surrogate of UV-B radiation exposure, leisure-time physical activity as a surrogate of sunlight exposure, adiposity, and dietary and supplementary vitamin D intake). In previous analyses, the score was correlated with plasma 25(OH)D levels and was significantly associated with the risk of developing colorectal cancer (Giovannucci *et al*, 2006). To assess the influence of post-diagnosis vitamin D status on colorectal cancer survival, we calculated the post-diagnosis 25(OH)D score among 1017 patients participating in two ongoing prospective cohort studies, the Nurses' Health Study (NHS) and the Health Professionals Follow-up Study (HPFS).

## Materials and methods

### Study population

In 1976, the NHS was established when 121 700 US female registered nurses aged 30–55 years answered a questionnaire on risk factors for cancer and cardiovascular disease (Belanger *et al*, 1978; Colditz *et al*, 1997). Every 2 years, participants receive follow-up questionnaires to update information on the potential risk factors and new disease diagnoses. Dietary information was first collected in 1980 and is updated in alternate follow-up cycles. Blood samples were provided by 32 826 participants aged 43–70 years from 1989 to 1990.

In 1986, the HPFS was established when 51 529 male dentists, optometrists, osteopaths, podiatrists, pharmacists, and veterinarians aged 40–75 years responded to a questionnaire on risk factors for cancer, cardiovascular disease, and diabetes. A follow-up questionnaire is sent to participants every 2 years requesting an update on non-dietary exposures and medical history, with dietary history updated every 4 years. Blood samples were provided by 18 018 participants from 1993 to 1995.

This study was approved by the Human Subjects Committee at Brigham and Women's Hospital and the Harvard School of Public Health in Boston, MA, USA. All participants provided informed consent for questionnaire and blood data to be used in research studies.

### Identification of colorectal cancer

On each follow-up questionnaire, participants were asked whether they had a diagnosis of colorectal cancer during the previous 2 years. When a participant (or next of kin for decedents) reported a diagnosis of colorectal cancer, we asked permission to obtain hospital records and pathology reports, and blinded study physicians reviewed and recorded information on tumour characteristics. For non-respondents, we searched the National Death Index to discover deaths and ascertain any diagnosis of colorectal cancer that contributed to death. We estimate that 96–97% of cases were identified through these methods (Giovannucci *et al*, 1994, 2006).

Individuals in this analysis were participants with pathologically confirmed colorectal adenocarcinoma diagnosed between 1986 (when physical activity was first assessed) and 2004.

### Measurement of mortality

Participants in the study cohort were followed until death or 2006. Ascertainment of deaths included reporting by family or postal authorities, and names of persistent non-responders were searched in the National Death Index (Sathiakumar *et al*, 1998). More than 98% of deaths have been identified by these methods (Stampfer *et al*, 1984; Giovannucci *et al*, 2006).

Cause of death was assigned by blinded physicians. Colorectal cancer-specific survival was defined as the time from diagnosis to death from colorectal cancer. Overall survival was defined as the time from diagnosis to death as a result of any cause.

### Exposure assessment

Derivation of the 25(OH)D prediction score has been described previously (Giovannucci *et al*, 2006). Briefly, linear regression was performed on 1095 men in the HPFS who were free of diagnosed cancer at the time of blood draw and who had available plasma 25(OH)D levels measured by radioimmunoassay (Hollis, 1997). Race, geographic region, dietary vitamin D intake, body mass index (BMI), and physical activity were identified as independent predictors of plasma 25(OH)D levels. Using the predictors' regression coefficients (Table 1), a 25(OH)D score was calculated for each cohort member. The model was able to predict a wide range of 25(OH)D levels, from 9 to 36 ng ml^−1^. The mean actual circulating level of 25(OH)D for men in the highest decile of predicted 25(OH)D was 11 ng ml^−1^ higher (95% CI, 9–13) than that of men in the lowest decile (1 ng ml^−1^=2.496 nmol l^−1^).

To validate this model, a 25(OH)D score was calculated for an independent sample of 542 men in HPFS who also had available plasma 25(OH)D measurements (Giovannucci *et al*, 2006). The actual plasma level rose across increasing deciles of predicted 25(OH)D (*P* trend<0.001), and the difference in the mean actual 25(OH)D level between extreme deciles was 10 ng ml^−1^, similar to the difference of 11 ng ml^−1^ in the initial dataset. Finally, in a separate analysis of 47 800 men, a statistically significant inverse association was observed between predicted 25(OH)D levels and the risk of developing colorectal cancer (Giovannucci *et al*, 2006).

Predicted 25(OH)D levels were calculated for our study cohort using the regression coefficients in Table 1 and extrapolating them to women. Post-diagnosis 25(OH)D score was the primary exposure variable, calculated using baseline race and geographic region, and values of physical activity, BMI, and vitamin D intake reported 1–4 years after colorectal cancer diagnosis (to avoid assessment during the period of active cancer treatment). Scores were divided into quintiles, with cut-offs determined separately for each cohort. We also examined the influence of pre-diagnosis predicted 25(OH)D levels, using values of physical activity, BMI, and vitamin D intake from the questionnaire administered immediately before diagnosis. If a response was missing, one previous assessment was carried forward; else, the patient was not included.

### Covariates

Prognostic factors known to influence cancer mortality were extracted from the medical record, including age, stage, grade of differentiation, tumour location, and year of diagnosis. In addition, BMI, physical activity, and total energy-adjusted calcium intake were taken from the questionnaire administered immediately before diagnosis. Race and season of diagnosis were also evaluated, but not found to be confounding variables, and were therefore excluded from the multivariable model. All analyses were adjusted for the cohort.

### Statistical analyses

Death from colorectal cancer was the primary end point, and death from any cause was a secondary end point. The primary statistical analysis used the two-tailed linear test for trend with predicted 25(OH)D level as a continuous variable, consistent with earlier studies (Feskanich *et al*, 2004), to avoid the possibility of selecting cut points with maximal *P*-values. To facilitate the display of our results, predicted 25(OH)D levels were defined in quintiles. Cox proportional hazards models were used to calculate hazard ratios (HRs) of death, adjusted for other risk factors for cancer survival. The HRs were calculated according to quintiles of predicted 25(OH)D levels, with the lowest quintile as the reference group. The proportionality of hazards assumption for the effect of predicted 25(OH)D level was satisfied by examining it as a time-dependent covariate in the Cox model.

Colorectal cancer-specific and overall mortality by tertile of predicted 25(OH)D levels were examined using Kaplan–Meier curves and the log-rank test (Therneau and Grambsch, 2000). Tertiles were used instead of quintiles for ease of graphical viewing.

Subgroup analyses were performed, and adjusted HRs and 95% CIs for an increment of 10 ng ml^−1^ of predicted 25(OH)D levels for cancer-specific and overall mortality were reported. Tests of interaction between predicted 25(OH)D levels and relevant covariates were assessed by entering in the model the cross product of predicted 25(OH)D level as a continuous variable with the covariate as a continuous or binary variable. All analyses used SAS software, version 9.1 (SAS Institute, Inc., Cary, NC, USA).

## Results

Among 1017 eligible participants, there were 283 deaths, 119 of which were due to colorectal cancer. The median time of follow-up of participants who were still alive was 116 months (range 41–238 months). The median predicted 25(OH)D level was 27.18 ng ml^−1^ in NHS and 29.18 ng ml^−1^ in HPFS. This pattern is consistent with what we saw in our previous analysis of pre-diagnosis plasma 25(OH)D levels and colorectal cancer survival, in which circulating 25(OH)D concentrations were also slightly higher in men than in women: median plasma 25(OH)D level was 27.1 ng ml^−1^ in men and 23.9 and 25.7 ng ml^−1^ for two laboratory runs in women (Ng *et al*, 2008). The difference in mean post-diagnosis predicted 25(OH)D levels between the highest and lowest deciles in this study cohort was 10 ng ml^−1^, which is similar to the difference in mean actual circulating 25(OH)D levels across extreme deciles of predicted 25(OH)D in the original and validation cohorts.

Baseline characteristics according to quintiles of post-diagnosis predicted 25(OH)D levels are shown in Table 2. Overall, participants with higher predicted 25(OH)D levels were more likely to be of white race, have a lower BMI, report higher physical activity, have higher calcium intake, and were more likely to be diagnosed with colorectal cancer in the summer or autumn. Other prognostic characteristics did not differ significantly between quintiles.

**Figure 1:**
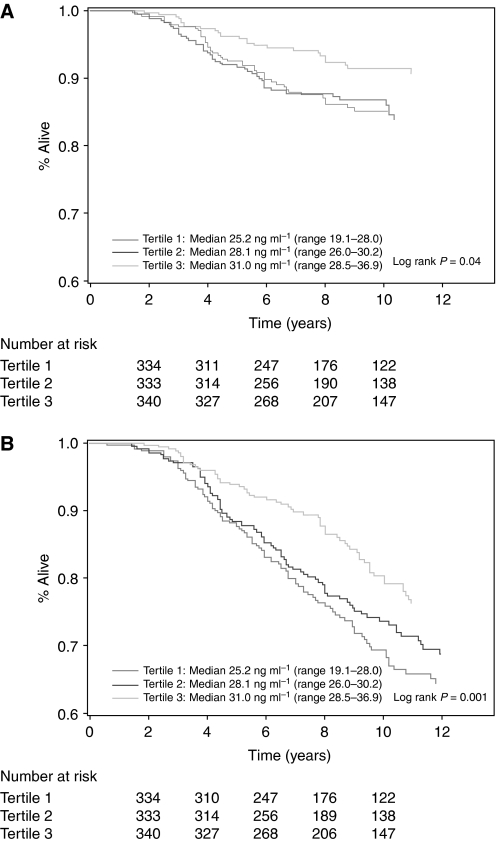
(**A**) Colorectal cancer-specific survival according to tertile of post-diagnosis predicted 25(OH)D levels. (**B**) Overall survival according to tertile of post-diagnosis predicted 25(OH)D levels.

Higher post-diagnosis predicted 25(OH)D levels were associated with a significant reduction in colorectal cancer-specific and overall mortality (Figure 1A and B, respectively, and Table 3). This relationship remained largely unchanged after adjusting for other predictors of cancer survival (Table 3). Compared with patients with post-diagnosis 25(OH)D scores in the lowest quintile, those in the highest quintile had an adjusted HR of 0.50 (95% CI, 0.26–0.95; *P* trend=0.02) for cancer-specific mortality and 0.62 (95% CI, 0.42–0.93; *P* trend=0.002) for overall mortality.

We considered the possibility that the 25(OH)D score may be acting as a surrogate for the causal factor, such as BMI or physical activity, which are both in the prediction equation. We therefore repeated our analyses after adjusting for BMI and physical activity. When BMI was included, the significant relationship between post-diagnosis 25(OH)D score and cancer-specific and overall mortality did not change. The adjusted HR was 0.51 (95% CI, 0.26–0.99; *P* trend=0.04) for cancer-specific mortality and 0.62 (95% CI, 0.41–0.94; *P* trend=0.005) for overall mortality, comparing extreme quintiles. Similarly, when we controlled for physical activity, we continued to observe a benefit for higher post-diagnosis predicted 25(OH)D levels, with an adjusted HR of 0.44 (95% CI, 0.22–0.87; *P* trend=0.01) for cancer-specific mortality and 0.67 (95% CI, 0.44–1.00; *P* trend=0.02) for overall mortality. When both BMI and physical activity were included in the model, the adjusted HR was 0.45 (95% CI, 0.22–0.91; *P* trend=0.02) for cancer-specific mortality and 0.66 (95% CI, 0.43–1.03; *P* trend=0.03) for overall mortality.

Moreover, when both BMI and physical activity were included in our model, the remaining components of the post-diagnosis 25(OH)D score that were not ‘accounted for’ were region of residence, race, and dietary and supplemental vitamin D intake. We therefore explored the impact of each of these individual variables on mortality in our study cohort, and found that patients who reported higher total vitamin D intake showed a trend towards lower risk of death (*P* trend=0.08). Compared with those in the lowest quintile, patients in the highest quintile of vitamin D intake had an adjusted HR of 0.72 (95% CI, 0.49–1.04) for overall mortality.

We also adjusted for calcium intake in our models and found a persistent significant association between post-diagnosis predicted 25(OH)D levels and survival. The adjusted HR was 0.44 (95% CI, 0.23–0.87; *P* trend=0.008) for cancer-specific mortality and 0.56 (95% CI, 0.37–0.84; *P* trend=0.0005) for overall mortality, comparing extreme quintiles. Of note, the addition of race and season of diagnosis to the multivariable model also did not change the adjusted HRs for cancer-specific and overall mortality. When race was added to the model, the adjusted HR comparing extreme quintiles was 0.53 (95% CI, 0.28–1.02; *P* trend=0.04) for cancer-specific mortality and 0.65 (95% CI, 0.43–0.97; *P* trend=0.004) for overall mortality. When season of diagnosis was included, the adjusted HR was 0.49 (95% CI, 0.26–0.94; *P* trend=0.02) for cancer-specific mortality and 0.63 (95% CI, 0.42–0.93; *P* trend=0.004) for overall mortality.

Given that lower levels of post-diagnosis predicted 25(OH)D could reflect the presence of occult cancer or other major illness, we excluded patients who died within 6 months of their post-diagnosis assessment. We continued to observe significant reductions in the risk of cancer-specific and overall mortality with increasing post-diagnosis 25(OH)D scores, with participants in the highest quintile having an adjusted HR of 0.50 (95% CI, 0.25–0.98; *P* trend=0.03) for cancer-specific mortality and 0.63 (95% CI, 0.42–0.95; *P* trend=0.003) for overall mortality.

In a separate analysis, pre-diagnosis 25(OH)D scores were calculated for colorectal cancer patients with available information (*n*=1955). Higher pre-diagnosis 25(OH)D scores were found to be associated with a decrease in cancer-specific and overall mortality (*P* trend=0.03 and 0.01, respectively). When we adjusted for pre-diagnosis predicted 25(OH)D levels as well as other predictors of cancer survival in our model, higher post-diagnosis predicted 25(OH)D levels were still associated with a significant reduction in both cancer-specific (*P* trend=0.02) and overall (*P* trend=0.008) mortality, whereas the effect of pre-diagnosis predicted 25(OH)D level was no longer significant.

**Figure 2:**
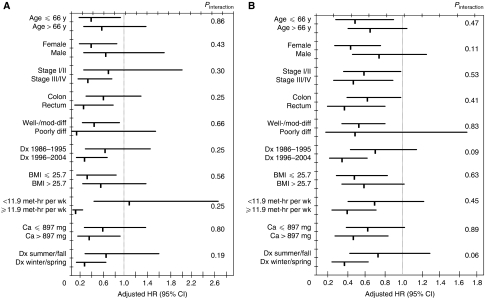
(**A**) Adjusted hazard ratios (HR) and 95% confidence intervals (CI) for an increment of 10 ng ml^−1^ of predicted 25(OH)D levels for colorectal cancer-specific mortality across strata of various factors. Mod-diff, moderately differentiated; Dx, diagnosed; BMI, body mass index (kg m^−2^); met, metabolic equivalents; hr, hours; wk, week; Ca, calcium; mg, milligrams. (**B**) Adjusted HR and 95% CI for an increment of 10 ng ml^−1^ of predicted 25(OH)D levels for overall mortality across strata of various factors. BMI, body mass index (kg m^−2^); Ca, calcium; Dx, diagnosed; hr, hours; met, metabolic equivalents; mg, milligrams; Mod-diff, moderately differentiated; wk, week; y, years.

We examined the influence of post-diagnosis 25(OH)D scores across the other predictors of cancer mortality (Figure 2A and B). Stratified analyses of cancer-specific and overall survival showed no significant interactions; the inverse relationship between post-diagnosis predicted 25(OH)D levels and mortality remained largely unchanged across most subgroups. Of note, there was a trend towards a greater impact of higher post-diagnosis 25(OH)D scores on overall survival in patients diagnosed in the winter or spring compared with those diagnosed in summer or autumn (*P* interaction=0.06).

## Discussion

Among patients with colorectal cancer, higher post-diagnosis predicted 25(OH)D levels were associated with a significant reduction in cancer-specific and overall mortality. This relationship was evident even after excluding patients who died within 6 months of their post-diagnosis 25(OH)D assessment, and across different subgroups of patients.

Previous studies have suggested an inverse relationship between vitamin D and cancer incidence. Prospective observational studies showed that higher plasma 25(OH)D levels are associated with a significant reduction in risk of colorectal cancer (Garland *et al*, 1989; Braun *et al*, 1995; Tangrea *et al*, 1997; Feskanich *et al*, 2004; Wactawski-Wende *et al*, 2006; Wu *et al*, 2007). In a prospective, placebo-controlled trial of vitamin D and calcium supplementation in 1179 women, a statistically significant decrease of 60% in all-cancer risk (including colorectal cancer) was seen in the intervention arm (*P*<0.03) (Lappe *et al*, 2007).

We have previously shown that higher pre-diagnosis plasma levels of 25(OH)D are associated with a significant 48% reduction in overall mortality (Ng *et al*, 2008). However, we only had a single measurement of plasma 25(OH)D levels taken at a median of 6 years before diagnosis. Furthermore, only 304 patients had plasma available for analysis. Therefore, to assess the influence of vitamin D status after cancer diagnosis in a larger population, we used a validated prediction score to estimate 25(OH)D levels after colorectal cancer diagnosis. This score is a reasonable predictor of circulating 25(OH)D levels, and is associated with cancer incidence and mortality (Giovannucci *et al*, 2006).

There are several mechanisms through which vitamin D may influence survival after a diagnosis of colorectal cancer. Binding of VDR by 1,25(OH)_2_D leads to transcriptional activation and repression of target genes, resulting in differentiation and apoptosis, and inhibition of proliferation and angiogenesis (reviewed by Deeb *et al*, 2007). *In vitro* and *in vivo* data have shown growth inhibition, and differentiation of colon carcinoma cell lines and xenografts by administration of 1,25(OH)_2_D (Eisman *et al*, 1987; Giuliano *et al*, 1991; Zhao and Feldman, 1993; Shabahang *et al*, 1994; Iseki *et al*, 1999), and rat models of colorectal cancer maintained on a 1,25(OH)_2_D diet developed fewer metastases compared with controls (Evans *et al*, 2000).

We found that season of diagnosis may modify the effect of predicted 25(OH)D levels on overall mortality, with participants diagnosed in the winter or spring showing a slightly greater reduction in the risk of death. Perhaps the impact of higher vitamin D levels is greater in the winter and spring, when sunlight exposure is at a minimum. For example, a patient with colorectal cancer with a high post-diagnosis 25(OH)D score, despite decreased UV-B exposure in winter and spring, may have a survival advantage over a patient with a low score in these seasons.

The strengths of our study include its prospective design, use of a validated prediction score, data on many potential confounders, and excellent follow-up rate. As participants were health professionals, the accuracy of self-reported data is likely to be high. To our knowledge, this study is the first to examine colorectal cancer survival by use of a comprehensive assessment of factors that determine 25(OH)D level. The similarity of our findings for survival based on one measurement of plasma 25(OH)D level and those based on our predictor score indicates that each may provide comparable information on long-term 25(OH)D level, the presumed factor of interest.

The most apparent limitation of our approach is the possibility that our 25(OH)D score is acting as a surrogate for the causal factor, such as BMI or physical activity, through alternative mechanisms. Physical activity has previously been shown to be associated with improved outcomes in colorectal cancer patients (Meyerhardt *et al*, 2006a, 2006b). In contrast, the data for BMI and colorectal cancer survival is conflicting (Meyerhardt *et al*, 2008). However, our results for predicted 25(OH)D levels did not change when adjusted for BMI or physical activity, and no significant interactions were observed. Moreover, when both BMI and physical activity were included in our model, examination of the remaining components of the post-diagnosis 25(OH)D score revealed that patients who reported higher total vitamin D intake showed a trend towards a lower risk of death. As dietary sources of vitamin D account for only 10% of circulating 25(OH)D levels (Adams *et al*, 1982), it is not unexpected that the finding is bordered on significance. This lends further support to the ability of our score to represent vitamin D status, and to our hypothesis that higher total vitamin D status – rather than simply adiposity or physical activity – is driving the significant association with survival. Furthermore, there is evidence that calcium may have an independent role in colorectal cancer pathogenesis (Wu *et al*, 2002; McCullough *et al*, 2003; Norat and Riboli, 2003). As the vitamin D pathway is intimately linked to calcium homoeostasis, we controlled for calcium intake in our analyses and found that our results did not change.

Although the 25(OH)D score had been developed and validated extensively in our cohort of men (Giovannucci *et al*, 2006), we recognise that the score has not been validated in women. However, there are no data in the current literature to suggest that the determinants of serum 25(OH)D differ between men and women. Furthermore, when our results were stratified by gender, similar HRs were obtained for men and women for both cancer-specific and overall mortality, with no significant interactions (see Figure 2A and B). Indeed, when post-diagnostic predicted 25(OH)D level was evaluated solely in the 606 women with colorectal cancer, a significant association was found between predicted 25(OH)D level and cancer-specific (adjusted HR 0.26; 95% CI, 0.10–0.72; *P* trend=0.02) and overall mortality (adjusted HR 0.48; 95% CI, 0.27–0.88; *P* trend=0.003). Moreover, any impression in the 25(OH)D score among women in our study would bias results in that cohort tend towards the null.

We also cannot completely exclude the possibility that lower levels of post-diagnosis predicted 25(OH)D reflect other occult predictors for poor prognosis. However, our findings remained unchanged after adjusting for potential risk factors of colorectal cancer mortality. To minimise bias in the post-diagnosis predicted 25(OH)D level by the presence of occult cancer or other major illness, we excluded patients who died within 6 months of their post-diagnosis vitamin D assessment, and continued to observe a positive impact of higher predicted 25(OH)D scores.

In this cohort, data on treatment were limited. Approximately half of the participants had stage I or II disease, for which surgery alone is often the standard of care. In addition, although there have been changes in the chemotherapeutic treatment of colorectal cancer during the timeframe under study, we adjusted for year of diagnosis in our models. Furthermore, the fairly homogeneous socioeconomic and educational makeup of this cohort likely minimises any disparities in chemotherapy receipt (VanEenwyk *et al*, 2002). The NHS and HPFS are composed exclusively of working health professionals with extensive access to health care; as such, differential access to state-of-the-art health care among participants is likely minimised. Lastly, because this was an observational rather than a randomised study, we cannot definitively attribute our results to 25(OH)D levels; further support for a causal role of higher vitamin D status on survival may require a randomised placebo-controlled trial.

In conclusion, our data suggest that higher levels of predicted 25(OH)D after a diagnosis of colorectal cancer may be associated with improved survival. Additional efforts to understand the mechanisms through which the vitamin D pathway influences colorectal carcinogenesis and cancer progression are warranted.

## Figures and Tables

**Table 1:** Factors contributing to predictors of age-adjusted plasma 25(OH)D level from a multiple linear regression model of 1095 men in the Health Professionals Follow-Up Study (Giovannucci *et al*, 2006)

**Factor**	**Change in 25(OH)D (ng ml^−1^**)
Intercept	33.81
	
*Race*	
White	0 (referent)
African American	−5.31
Asian	−5.49
Other	−0.17
	
*Residence*	
Northeast/Mid-Atlantic	0 (referent)
Midwest/West	+1.61
South	+2.56
	
*Quintile of leisure-time physical activity (MET-hr per week)* a	
5	0 (referent)
4	−1.81
3	−3.07
2	−3.59
1	−5.40
	
*Body mass index (kg m*^−*2*^)	
<22	0 (referent)
22–24.9	−0.40
25–29.9	−1.80
30–34.9	−2.58
⩾35	−3.44
	
*Dietary vitamin D (IU per day)*	
⩾400	0 (referent)
300–399	−1.39
200–299	−1.04
100–199	−2.85
<100	−4.16
	
*Supplementary vitamin D (IU per day)* b	
⩾400	0 (referent)
200–399	−0.71
1–199	+0.97
<1	−0.83
	
*Season of blood draw* c	
Autumn (September, October, and November)	0 (referent)
Summer (June, July, and August)	−0.74
Spring (March, April, and May)	−4.83
Winter (December, January, and February)	−5.42

Abbreviations: 25(OH)D=25-hydroxyvitamin D_3_; hr=hour; IU=international units; MET=metabolic equivalents.

a Physical activity is used as a proxy for outdoor activities, which will tend to increase solar UV-B exposure.

b Not statistically significant.

c Season of blood draw was adjusted but not used in the predictive model because season is a strong determinant of 25(OH)D level and reflects the time of blood draw, but it is not a factor in determining long-term average between-person variation in 25(OH)D level.

**Table 2:** Baseline characteristics of cohort according to quintile of predicted 25(OH)D (*n*=1017)

	**Predicted 25(OH)D (ng ml^−1^)**					
**Characteristic**	**Quintile 1**	**Quintile 2**	**Quintile 3**	**Quintile 4**	**Quintile 5**	***P*-value**
No. of patients	204	202	205	203	203	—
Median predicted 25(OH)D (ng ml^−1^)^a^ – NHS (*n*=606)	23.3 (range 19.1–24.8)	25.7 (range 24.9–26.4)	27.2 (range 26.5–28.0)	28.6 (range 28.0–29.7)	31.0 (range 29.7–34.9)	—
Median predicted 25(OH)D, (ng ml^−1^)^a^ – HPFS (*n*=411)	25.8 (range 21.6–26.8)	27.8 (range 27.0–28.4)	29.2 (range 28.5–29.9)	30.8 (range 29.9–31.5)	32.8 (range 31.5–36.9)	—
Mean age at diagnosis (years)	65.6 (s.e. 0.6)	66.0 (s.e. 0.5)	65.8 (s.e. 0.6)	65.7 (s.e. 0.5)	65.3 (s.e. 0.5)	0.92^b^
						
*Stage* (%)						0.95^c^
I	33	30	34	34	34	
II	28	30	25	30	32	
III	23	25	26	22	21	
IV	4	4	2	4	3	
Unknown	12	11	13	10	10	
						
*Grade of tumor differentiation* (%)						0.65^c^
Well differentiated	12	14	12	11	19	
Moderately differentiated	59	58	58	60	57	
Poorly differentiated	11	10	12	11	11	
Unknown	18	18	18	18	13	
						
*Location of primary tumor* (%)						0.43^c^
Proximal colon	36	38	39	40	41	
Distal colon	33	37	33	35	31	
Rectum	22	22	23	19	24	
Unknown	9	3	5	6	4	
						
*Year of diagnosis* (%)						0.32^c^
1986–1995	47	46	52	49	55	
1996–2004	53	54	48	51	45	
						
*Season of diagnosis*^d^ (%)						0.003^c^
Summer	30	27	32	31	23	
Autumn	20	23	20	34	26	
Winter	25	22	27	19	23	
Spring	25	28	21	16	28	
						
*Race* (%)						<0.0001^c^
White	89	93	95	98	95	
Black	5	1	0	0	0	
Other	6	6	5	2	5	
Mean body mass index at diagnosis (kg m^−2^)	28.7 (s.e. 0.3)	27.3 (s.e. 0.3)	26.1 (s.e. 0.3)	25.0 (s.e. 0.2)	24.4 (s.e. 0.2)	<0.0001^b^
Median physical activity at diagnosis (MET-hr per week)^e^	5.3 (range 0–148.1)	8.4 (range 0–98.0)	12.9 (range 0–131.8)	15.4 (range 0–127.7)	23.0 (range 0–625.2)	<0.0001^f^
Median total energy-adjusted calcium intake (mg)^e^	786 (range 248–3446)	817 (range 260–3375)	930 (range 329–3192)	936 (range 239–2935)	1010 (range 346–4496)	<0.0001^f^

Abbreviations: 25(OH)D=25-hydroxyvitamin D_3_; HPFS=Health Professionals Follow-up Study; hr=hour; IU=international units; MET=metabolic equivalents; NHS=Nurses' Health Study; No.=number; s.e.=standard error.

a 1 ng ml^−1^=2.496 nmol l^−1^.

b *P*-value calculated using one-way analysis of variance.

c *P*-value calculated using *χ*^2^-test.

d Summer defined as June, July, and August; autumn defined as September, October, and November; winter defined as December, January, and February; spring defined as March, April, and May.

e Analysis restricted to those participants with available information.

f *P*-value calculated using Kruskal–Wallis test.

**Table 3:** Age-adjusted and multivariate hazard ratios of death according to quintile of predicted 25(OH)D in the entire study cohort (*n*=1017)

	**Predicted 25(OH)D**										
	**Quintile 1**		**Quintile 2**		**Quintile 3**		**Quintile 4**		**Quintile 5**		
**Variable**	**No./ event**	**HR (95% CI)**	**No./ event**	**HR (95% CI)**	**No./ event**	**HR (95% CI)**	**No./ event**	**HR (95% CI)**	**No./ event**	**HR (95% CI)**	***P*-trend^a^**
*Colorectal cancer-specific mortality*											
Age-adjusted^b^	204/29	Referent	202/29	1.02 (0.61–1.70)	205/28	0.95 (0.56–1.59)	203/19	0.62 (0.35–1.10)	203/14	0.45 (0.24–0.84)	0.006
Multivariate^c^				0.99 (0.58–1.68)		1.04 (0.61–1.78)		0.62 (0.34–1.11)		0.50 (0.26–0.95)	0.02
											
*Overall mortality*											
Age-adjusted^b^	204/64	Referent	202/71	1.13 (0.80–1.58)	205/62	0.96 (0.68–1.36)	203/44	0.62 (0.42–0.91)	203/42	0.57 (0.38–0.84)	0.0004
Multivariate^c^				1.19 (0.85–1.68)		1.05 (0.74–1.50)		0.63 (0.43–0.94)		0.62 (0.42–0.93)	0.002

Abbreviations: 25(OH)D=25-hydroxyvitamin D_3_; CI=confidence interval; HR=hazard ratio; No.=number.

a Calculated by using predicted 25(OH)D as a continuous variable, adjusted for cohort.

b HRs, 95% CIs, and *P*-values are adjusted for age at diagnosis (years).

c Multivariate HRs, 95% CIs, and *P*-values are adjusted for age at diagnosis (in years as a continuous variable), gender (male or female), cancer stage (I–IV or unknown), grade of tumor differentiation (well-differentiated, moderately-differentiated, poorly-differentiated, or unspecified/missing), location of primary tumor (proximal, distal, rectum, or unknown), and year of diagnosis (as a continuous variable).

## References

[bib1] Adams JS, Clemens TL, Parrish JA, Holick MF (1982) Vitamin-D synthesis and metabolism after ultraviolet irradiation of normal and vitamin-D-deficient subjects. N Engl J Med 306: 722–725 7038486 10.1056/NEJM198203253061206

[bib2] Belanger CF, Hennekens CH, Rosner B, Speizer FE (1978) The nurses' health study. Am J Nurs 78: 1039–1040 248266

[bib3] Braun MM, Helzlsouer KJ, Hollis BW, Comstock GW (1995) Colon cancer and serum vitamin D metabolite levels 10–17 years prior to diagnosis. Am J Epidemiol 142: 608–611 7653469 10.1093/oxfordjournals.aje.a117682

[bib4] Colditz GA, Manson JE, Hankinson SE (1997) The Nurses' Health Study: 20-year contribution to the understanding of health among women. J Womens Health 6: 49–62 9065374 10.1089/jwh.1997.6.49

[bib5] Deeb KK, Trump DL, Johnson CS (2007) Vitamin D signalling pathways in cancer: potential for anticancer therapeutics. Nat Rev Cancer 7: 684–700 17721433 10.1038/nrc2196

[bib6] Diaz GD, Paraskeva C, Thomas MG, Binderup L, Hague A (2000) Apoptosis is induced by the active metabolite of vitamin D3 and its analogue EB1089 in colorectal adenoma and carcinoma cells: possible implications for prevention and therapy. Cancer Res 60: 2304–2312 10786699

[bib7] Eisman JA, Barkla DH, Tutton PJ (1987) Suppression of *in vivo* growth of human cancer solid tumor xenografts by 1,25-dihydroxyvitamin D3. Cancer Res 47: 21–25 3024816

[bib8] Evans SR, Shchepotin EI, Young H, Rochon J, Uskokovic M, Shchepotin IB (2000) 1,25-dihydroxyvitamin D3 synthetic analogs inhibit spontaneous metastases in a 1,2-dimethylhydrazine-induced colon carcinogenesis model. Int J Oncol 16: 1249–1254 10812003 10.3892/ijo.16.6.1249

[bib9] Fernandez-Garcia NI, Palmer HG, Garcia M, Gonzalez-Martin A, del Rio M, Barettino D, Volpert O, Munoz A, Jimenez B (2005) 1Alpha,25-dihydroxyvitamin D3 regulates the expression of Id1 and Id2 genes and the angiogenic phenotype of human colon carcinoma cells. Oncogene 24: 6533–6544 16007183 10.1038/sj.onc.1208801

[bib10] Feskanich D, Ma J, Fuchs CS, Kirkner GJ, Hankinson SE, Hollis BW, Giovannucci EL (2004) Plasma vitamin D metabolites and risk of colorectal cancer in women. Cancer Epidemiol Biomarkers Prev 13: 1502–1508 15342452

[bib11] Garland CF, Comstock GW, Garland FC, Helsing KJ, Shaw EK, Gorham ED (1989) Serum 25-hydroxyvitamin D and colon cancer: eight-year prospective study. Lancet 2: 1176–1178 2572900 10.1016/s0140-6736(89)91789-3

[bib12] Giovannucci E, Colditz GA, Stampfer MJ, Hunter D, Rosner BA, Willett WC, Speizer FE (1994) A prospective study of cigarette smoking and risk of colorectal adenoma and colorectal cancer in US women. J Natl Cancer Inst 86: 192–199 8283491 10.1093/jnci/86.3.192

[bib13] Giovannucci E, Liu Y, Rimm EB, Hollis BW, Fuchs CS, Stampfer MJ, Willett WC (2006) Prospective study of predictors of vitamin D status and cancer incidence and mortality in men. J Natl Cancer Inst 98: 451–459 16595781 10.1093/jnci/djj101

[bib14] Giuliano AR, Franceschi RT, Wood RJ (1991) Characterization of the vitamin D receptor from the Caco-2 human colon carcinoma cell line: effect of cellular differentiation. Arch Biochem Biophys 285: 261–269 1654769 10.1016/0003-9861(91)90358-p

[bib15] Gorham ED, Garland CF, Garland FC, Grant WB, Mohr SB, Lipkin M, Newmark HL, Giovannucci E, Wei M, Holick MF (2007) Optimal vitamin D status for colorectal cancer prevention: a quantitative meta analysis. Am J Prev Med 32: 210–216 17296473 10.1016/j.amepre.2006.11.004

[bib16] Hollis BW (1997) Quantitation of 25-hydroxyvitamin D and 1,25-dihydroxyvitamin D by radioimmunoassay using radioiodinated tracers. Methods Enzymol 282: 174–186 9330287 10.1016/s0076-6879(97)82106-4

[bib17] Iseki K, Tatsuta M, Uehara H, Iishi H, Yano H, Sakai N, Ishiguro S(1999) Inhibition of angiogenesis as a mechanism for inhibition by 1alpha-hydroxyvitamin D3 and 1,25-dihydroxyvitamin D3 of colon carcinogenesis induced by azoxymethane in Wistar rats. Int J Cancer 81: 730–733 10328225 10.1002/(sici)1097-0215(19990531)81:5<730::aid-ijc11>3.0.co;2-q

[bib18] Lamprecht SA, Lipkin M (2001) Cellular mechanisms of calcium and vitamin D in the inhibition of colorectal carcinogenesis. Ann NY Acad Sci 952: 73–87 11795445 10.1111/j.1749-6632.2001.tb02729.x

[bib19] Lappe JM, Travers-Gustafson D, Davies KM, Recker RR, Heaney RP (2007) Vitamin D and calcium supplementation reduces cancer risk: results of a randomized trial. Am J Clin Nutr 85: 1586–1591 17556697 10.1093/ajcn/85.6.1586

[bib20] McCullough ML, Robertson AS, Rodriguez C, Jacobs EJ, Chao A, Carolyn J, Calle EE, Willett WC, Thun MJ (2003) Calcium, vitamin D, dairy products, and risk of colorectal cancer in the Cancer Prevention Study II Nutrition Cohort (United States). Cancer Causes Control 14: 1–12 12708719 10.1023/a:1022591007673

[bib21] Meggouh F, Lointier P, Saez S (1991) Sex steroid and 1,25-dihydroxyvitamin D3 receptors in human colorectal adenocarcinoma and normal mucosa. Cancer Res 51: 1227–1233 1847660

[bib22] Meyerhardt JA, Giovannucci EL, Holmes MD, Chan AT, Chan JA, Colditz GA, Fuchs CS (2006a) Physical activity and survival after colorectal cancer diagnosis. J Clin Oncol 24: 3527–3534 16822844 10.1200/JCO.2006.06.0855

[bib23] Meyerhardt JA, Heseltine D, Niedzwiecki D, Hollis D, Saltz LB, Mayer RJ, Thomas J, Nelson H, Whittom R, Hantel A, Schilsky RL, Fuchs CS (2006b) Impact of physical activity on cancer recurrence and survival in patients with stage III colon cancer: findings from CALGB 89803. J Clin Oncol 24: 3535–3541 16822843 10.1200/JCO.2006.06.0863

[bib24] Meyerhardt JA, Niedzwiecki D, Hollis D, Saltz LB, Mayer RJ, Nelson H, Whittom R, Hantel A, Thomas J, Fuchs CS (2008) Impact of body mass index and weight change after treatment on cancer recurrence and survival in patients with stage III colon cancer: findings from Cancer and Leukemia Group B 89803. J Clin Oncol 26: 4109–4115 18757324 10.1200/JCO.2007.15.6687 PMC2654367

[bib25] Ng K, Meyerhardt JA, Wu K, Feskanich D, Hollis BW, Giovannucci EL, Fuchs CS (2008) Circulating 25-hydroxyvitamin d levels and survival in patients with colorectal cancer. J Clin Oncol 26: 2984–2991 18565885 10.1200/JCO.2007.15.1027

[bib26] Norat T, Riboli E (2003) Dairy products and colorectal cancer. A review of possible mechanisms and epidemiological evidence. Eur J Clin Nutr 57: 1–17 12548291 10.1038/sj.ejcn.1601522

[bib27] Sathiakumar N, Delzell E, Abdalla O (1998) Using the National Death Index to obtain underlying cause of death codes. J Occup Environ Med 40: 808–813 9777565 10.1097/00043764-199809000-00010

[bib28] Scaglione-Sewell BA, Bissonnette M, Skarosi S, Abraham C, Brasitus TA (2000) A vitamin D3 analog induces a G1-phase arrest in CaCo-2 cells by inhibiting cdk2 and cdk6: roles of cyclin E, p21Waf1, and p27Kip1. Endocrinology 141: 3931–3939 11089522 10.1210/endo.141.11.7782

[bib29] Shabahang M, Buras RR, Davoodi F, Schumaker LM, Nauta RJ, Uskokovic MR, Brenner RV, Evans SR (1994) Growth inhibition of HT-29 human colon cancer cells by analogues of 1,25-dihydroxyvitamin D3. Cancer Res 54: 4057–4064 8033137

[bib30] Stampfer MJ, Willett WC, Speizer FE, Dysert DC, Lipnick R, Rosner B, Hennekens CH (1984) Test of the National Death Index. Am J Epidemiol 119: 837–839 6720679 10.1093/oxfordjournals.aje.a113804

[bib31] Tangrea J, Helzlsouer K, Pietinen P, Taylor P, Hollis B, Virtamo J, Albanes D (1997) Serum levels of vitamin D metabolites and the subsequent risk of colon and rectal cancer in Finnish men. Cancer Causes Control 8: 615–625 9242478 10.1023/a:1018450531136

[bib32] Therneau T, Grambsch P (2000) Modeling Survival Data. Springer: New York, NY

[bib33] Vandewalle B, Adenis A, Hornez L, Revillion F, Lefebvre J (1994) 1,25-dihydroxyvitamin D3 receptors in normal and malignant human colorectal tissues. Cancer Lett 86: 67–73 7954357 10.1016/0304-3835(94)90181-3

[bib34] Vandewalle B, Wattez N, Lefebvre J (1995) Effects of vitamin D3 derivatives on growth, differentiation and apoptosis in tumoral colonic HT 29 cells: possible implication of intracellular calcium. Cancer Lett 97: 99–106 7585485 10.1016/0304-3835(95)03958-y

[bib35] VanEenwyk J, Campo JS, Ossiander EM (2002) Socioeconomic and demographic disparities in treatment for carcinomas of the colon and rectum. Cancer 95: 39–46 12115315 10.1002/cncr.10645

[bib36] Wactawski-Wende J, Kotchen JM, Anderson GL, Assaf AR, Brunner RL, O'Sullivan MJ, Margolis KL, Ockene JK, Phillips L, Pottern L, Prentice RL, Robbins J, Rohan TE, Sarto GE, Sharma S, Stefanick ML, Van Horn L, Wallace RB, Whitlock E, Bassford T, Beresford SA, Black HR, Bonds DE, Brzyski RG, Caan B, Chlebowski RT, Cochrane B, Garland C, Gass M, Hays J, Heiss G, Hendrix SL, Howard BV, Hsia J, Hubbell FA, Jackson RD, Johnson KC, Judd H, Kooperberg CL, Kuller LH, LaCroix AZ, Lane DS, Langer RD, Lasser NL, Lewis CE, Limacher MC, Manson JE (2006) Calcium plus vitamin D supplementation and the risk of colorectal cancer. N Engl J Med 354: 684–696 16481636 10.1056/NEJMoa055222

[bib37] Wu K, Feskanich D, Fuchs CS, Willett WC, Hollis BW, Giovannucci EL (2007) A nested case control study of plasma 25-hydroxyvitamin D concentrations and risk of colorectal cancer. J Natl Cancer Inst 99: 1120–1129 17623801 10.1093/jnci/djm038

[bib38] Wu K, Willett WC, Fuchs CS, Colditz GA, Giovannucci EL (2002) Calcium intake and risk of colon cancer in women and men. J Natl Cancer Inst 94: 437–446 11904316 10.1093/jnci/94.6.437

[bib39] Zehnder D, Bland R, Williams MC, McNinch RW, Howie AJ, Stewart PM, Hewison M (2001) Extrarenal expression of 25-hydroxyvitamin d(3)-1 alpha-hydroxylase. J Clin Endocrinol Metab 86: 888–894 11158062 10.1210/jcem.86.2.7220

[bib40] Zhao X, Feldman D (1993) Regulation of vitamin D receptor abundance and responsiveness during differentiation of HT-29 human colon cancer cells. Endocrinology 132: 1808–1814 8384998 10.1210/endo.132.4.8384998

