# Prospective study of predictors of vitamin D status and survival in patients with colorectal cancer

**DOI:** 10.1038/sj.bjc.6605614

**Published:** 2010-03-16

**Authors:** K Ng, B M Wolpin, J A Meyerhardt, K Wu, A T Chan, B W Hollis, E L Giovannucci, M J Stampfer, W C Willett, C S Fuchs

**Correction to**: *British Journal of Cancer* (2009) **101**, 916–923; doi:10.1038/sj.bjc.6605262

After publication of the above paper in Volume 101, Number 6, the authors realised that they had intended to include an Acknowledgements paragraph in the article. The paragraph should read as follows:

